# Neurocognitive Adaptations for Spatial Orientation and Navigation in Astronauts

**DOI:** 10.3390/brainsci13111592

**Published:** 2023-11-15

**Authors:** Ford Burles, Giuseppe Iaria

**Affiliations:** 1 Canadian Space Health Research Network, Department of Psychology, Hotchkiss Brain Institute, Alberta Children’s Hospital Research Institute, University of Calgary, Calgary, AB T2N 1N4, Canada; giaria@ucalgary.ca; 2 NeuroLab, Department of Psychology, Hotchkiss Brain Institute, Alberta Children’s Hospital Research Institute, University of Calgary, Calgary, AB T2N 1N4, Canada

**Keywords:** brain, extreme environment, microgravity, parietal cortex, spaceflight

## Abstract

Astronauts often face orientation challenges while on orbit, which can lead to operator errors in demanding spatial tasks. In this study, we investigated the impact of long-duration spaceflight on the neural processes supporting astronauts’ spatial orientation skills. Using functional magnetic resonance imaging (fMRI), we collected data from 16 astronauts six months before and two weeks after their International Space Station (ISS) missions while performing a spatial orientation task that requires generating a mental representation of one’s surroundings. During this task, astronauts exhibited a general reduction in neural activity evoked from spatial-processing brain regions after spaceflight. The neural activity evoked in the precuneus was most saliently reduced following spaceflight, along with less powerful effects observed in the angular gyrus and retrosplenial regions of the brain. Importantly, the reduction in precuneus activity we identified was not accounted for by changes in behavioral performance or changes in grey matter concentration. These findings overall show less engagement of explicitly spatial neurological processes at postflight, suggesting astronauts make use of complementary strategies to perform some spatial tasks as an adaptation to spaceflight. These preliminary findings highlight the need for developing countermeasures or procedures that minimize the detrimental effects of spaceflight on spatial cognition, especially in light of planned long-distance future missions.

## 1. Introduction

The ability to orient and navigate spatial surroundings is critical for effective daily life functioning. In terrestrial environments, generating a coherent understanding of the environment and our movements within it depends on integrating information from different sensory modalities, combining what we see, the movement-related vestibular cues that we perceive, and the proprioceptive information that we process while locomoting [1,2,3,4]. Among these different sources of information, the Earth’s constant gravitational force allows neurophysiological and cognitive processes to develop with gravitational assumptions built in [5,6], facilitating the process of moving throughout the environment and constructing a mental representation for the purpose of spatial orientation and navigation. However, the typical cognitive frameworks and strategies we use when navigating spatial surroundings often fail when these assumptions no longer hold. This is the case for astronauts and cosmonauts who routinely report transient orientation and navigation difficulties while exposed to microgravity during a spaceflight [7,8,9,10].

The altered set of sensory information experienced in spaceflight environments poses a unique challenge for astronauts performing spatial tasks. In fact, upon their initial exposure to microgravity, the majority of astronauts report experiencing space motion sickness, triggered by the newly incoherent information from the vestibular system in comparison to the typically complementary visuospatial and proprioceptive information processed on Earth [11]. However, the vestibular system is highly adaptable [12], and these symptoms usually resolve to non-debilitating levels over a few days [11]. This adaptation has been ascribed to a reweighting or a transient multimodal disintegration of some vestibular signals [13,14]. Given the inherently multimodal nature of spatial processing in the brain [4,15,16], one would expect that these necessary adaptations to microgravity would alter how astronauts perform spatial tasks in microgravity, affecting the neural mechanisms that are responsible for spatial orientation and navigation.

One such neural mechanism, the head direction signal, has been shown to have vestibular underpinnings in both its generation and maintenance [17]. While this is not the only contribution vestibular information has on spatial processing generally [18], it is an important and salient contribution to spatial processing for navigation (as opposed to spatial processing for postural awareness, as an example). Indeed, the head direction signal, along with a neural representation of location, constitutes the two critical components—heading and position—needed for effective wayfinding and navigation. Vestibular information, originating from the semicircular canals and otolith organs, passes through a handful of nuclei before converging on the lateral mammillary and dorsal tegmental nuclei, the putative location at which the head direction signal is generated [17]. This information continues to propagate through the thalamus to the retrosplenial complex and entorhinal cortex, at which point they contribute to higher-level spatial associations and representations, generally thought to contribute more to abstracted, cognitive-map-like representations at these latter brain regions [19,20].

Here, we set out to characterize the manner in which long-duration spaceflight alters the neural underpinnings of spatial cognition in astronauts. We asked astronauts to perform a challenging spatial task—the spatial configuration task [21]—while we collected functional magnetic resonance imaging (fMRI) data before and after their missions onboard the ISS. The spatial configuration task required astronauts to integrate multiple viewpoints into a coherent mental representation of the environment and, subsequently, use that mental representation to infer their location based on limited visual information, a cognitive process that is known to support effective spatial orientation and navigation in humans [22]. In non-astronaut subjects, we have demonstrated that the spatial configuration task elicits robust fMRI activity from a network of cortical regions (e.g., the precuneus, lingual, fusiform, parahippocampal and retrosplenial complex) that are known to play a critical role in spatial orientation and navigation [21]. These brain regions are involved in processing spatial information from sensory inputs, performing spatial computations, and recalling spatial information from memory [23,24,25]. Given the well-known and salient alterations to many of the nonvisual sensory inputs and changes in motor affordances occurring in spaceflight, which are important to process spatial information [4,16,26,27,28,29], we hypothesized that exposure to microgravity will generally produce changes in brain activity in those regions that are critical for spatial updating and processing sensory information for effective orientation and navigation. Particularly, we anticipate the retrosplenial complex to show the most salient spaceflight-related functional perturbations, given its role in processing heading direction specifically [4] and linking perceptual information to a mental representation of space more generally [30].

## 2. Materials and Methods

### 2.1. Participants

We collected data from 16 astronauts as part of the Canadian Space Agency (CSA-ASC)-funded “Wayfinding” project. This sample included 7 women and 9 men, with an *M* (*SD*) age of 45.73 (5.70) years at preflight testing who participated in typical ISS missions with a duration of 204.21 (44.01) days. This sample had an average previous spaceflight experience *M* (*SD*) of 48.48 (71.02) days, across 0.71 (0.81) flights. We analyzed structural and functional MRI data collected from these participants 219.21 (118.35) days before launch and 12.43 (1.82) days after landing.

### 2.2. MRI Acquisition

We collected structural and functional MRI data from a 3T Siemens Verio running Syngo MR B19 using a 32-channel head coil at the University of Texas Medical Branch (UTMB) Victory Lakes facility in League City, TX, USA. For each timepoint, structural acquisitions included a 3 min 45 s magnetization-prepared rapid acquisition gradient echo (MPRAGE) scan (2.3 s repetition time, 2.34 ms echo time, 8° flip angle, GRAPPA factor of 3) and a 5 min 52 s fluid-attenuated inversion recovery (FLAIR) scan (5 s repetition time, 281 ms echo time, GRAPPA factor of 3), both sagittal acquisitions with 1 mm isotropic voxels. Functional acquisitions included two runs of a 6 min 30 s Blood Oxygen Level Dependent (BOLD) echo planar imaging sequence (2.52 s repetition time, 28 ms echo time, 80° flip angle, axial acquisition with 3.4 × 3.4 × 3.2 mm^3^ voxels, GRAPPA factor of 3).

### 2.3. MRI Task Design

During each functional run, astronauts performed three minutes (i.e., 20 trials) of both a spatial task and a control task, the order of which was randomly selected on a per-run basis. Tasks were separated with 12 s of a fixation cross and prepended with 2 s of a brief task reminder. Tasks were created and presented using Presentation^®^ (Neurobehavioral Systems, Inc., Albany, CA, USA). Participants were familiarized with the tasks before scanning began. Due to acquisition time constraints, we shortened the trial time of the “spatial configuration task” and non-spatial control task utilized in previous work [21], by reducing the time after participants’ response and before the camera moved to a new trial when the camera was merely “waiting” stationary. This modification resulted in shorter trials, reducing our capacity to contrast the different phases of the task, as we have done in our previous work.

**Figure 1:**
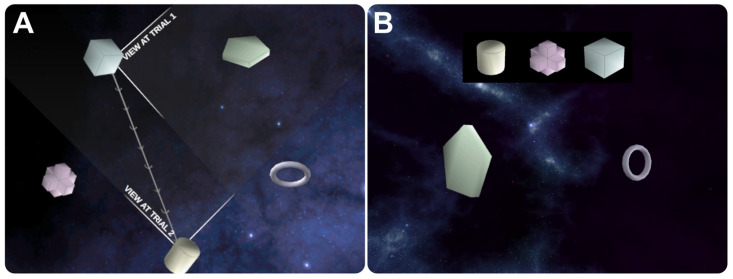
The spatial configuration task and control task performed while we collected fMRI data from astronauts. The top-down view of a sample environment shown in Panel (**A**) depicts an example of how the camera would move from trial to trial in both tasks. Panel (**B**) depicts the participant’s perspective at a trial in either task, with the objects depicted at the top of the screen, indicating the response options available to the participant. Participants never see the environment from the perspective shown in Panel (**A**).

In both tasks, five simple geometric objects are arranged pseudorandomly in a space-like virtual environment (Figure 1A). At each trial of the spatial task, participants view two objects of the environment from the perspective of a third object. Participants are tasked with identifying which object they are looking from, i.e., which object the camera is positioned upon, from three response options that include all environmental objects not currently visible (Figure 1B). After the response phase, there is a brief pause, and the camera moves to a new object, again viewing another pair of objects, and the next trial begins. This requires participants to generate a mental representation of the environment over successive trials and use that mental representation to determine their location from the viewed scene at each trial.

In each trial of the control task, participants view the same type of stimuli, except they are tasked with identifying which object they did not see in the environment at the previous trial (i.e., a modified one-back task). For instance, in the example trial pair depicted in Figure 1A, during the first trial, the participant viewed the pentagon and ring, and in the following trial, they would be presented with the pentagon and ring as two of the three response options and would need to select the third option. The control task has equivalent visual stimuli and expected response patterns as compared to the spatial task but does not have any explicit spatial processing demands. In the control task, the positions of unseen objects are swapped at each trial to prevent any implicit spatial mapping that might occur. Video instructions were provided to participants to ensure they understood both the spatial [31] and control [32] tasks before data collection and are publicly available at the referenced URLs.

### 2.4. MRI Processing

We prepared each subject’s MRI data for analysis with the following steps:

1. Using the Statistical Parametric Mapping (SPM12, Wellcome Centre for Human Neuroimaging, London, England, UK, 2018) software version 7487, we independently brain-extracted the MPRAGE and FLAIR data using the unified segmentation module. Default parameters were used, except bias-field-removed images were output, sampling distance was set to 2.5 mm, and n Gaussians for tissue classes 1 and 2 (i.e., grey and white matter) were set to 2.
2. We generated brain masks for both MPRAGE and FLAIR images by combining the grey matter, white matter, and cerebrospinal fluid segmentation results from (1) using *fslmaths*, ‘FSL’ version 6.0.6, (FMRIB Analysis Group, Oxford University, Oxford, UK, 2022). We binarized the combined data, smoothed it with a 2 mm sigma Gaussian kernel, and re-binarized it at a threshold of 0.75.
3. We used the antsRegistration.sh script from ANTs version 2.4.1 to non-linearly warp the bias-field-corrected and non-brain-extracted MPRAGE image from (1) to the bias-field-corrected and non-brain-extracted FLAIR image from (1). This registration included a rigid-body mutual information registration, followed by a heavily regularized SyN deformation [33] to ensure the resulting warps were spatially smooth. This was performed to correct for deformations between the MPRAGE and FLAIR images resulting from their differing receiver bandwidths. The FLAIR image was selected to define the resultant space because its higher bandwidth results in less geometric distortion. Both registrations were computed using masks from (2).
4. We then performed a fine multimodal segmentation [34] and normalization using SPM12’s unified segmentation [35] with the bias-field-corrected FLAIR from (1) and the warped and bias-field-corrected MPRAGE from (3). Default parameters were used, except additionally, we output forward warps to Montreal Neurological Institute (MNI) space, using a sampling distance of 1 mm and setting the n Gaussians to 2, 2, 3, 4 and 5, for tissue classes 1 through 5, respectively.
5. We motion-corrected the functional MRI data from each run in SPM12 using the Realign module. Default parameters were used except estimation quality was set to 0.95, separation was set to 3 mm, and smoothing was set to 4 mm full width at half maximum (FWHM), using a 3rd-degree B-spline for estimation interpolation. These data, as well as a mean image, were resliced using a 6th-degree B-spline.
6. We performed slice-time correction on the motion-corrected outputs from (5) using SPM12 with the reference slice set at the spatial center of the acquisition volume.
7. We coregistered the mean volume from (5) to the bias-field-corrected FLAIR image in SPM12’s coregistration module. We used default parameters, except set the estimation separation to (3 mm, 1 mm). We carried slice-time-corrected outputs from (6) along the computed transformation.
8. We moved the coregistered slice-time-corrected fMRI data from (7) and the native space grey matter, white matter, and CSF tissue map from (4) to 2 mm isotropic MNI space using SPM12’s Normalize module with warps computed from (4). Data from (7) were interpolated with a 7th-degree B-spline, and the tissue maps for classes grey matter, white matter, and CSF from (4) were interpolated using trilinear interpolation.
9. We computed additional first-level fMRI regressors with an in-house Python script. The resulting set of first-level fMRI regressors included six motion regressors, their temporal derivatives, framewise displacement, scrubbing regressors for global signal spikes calculated from (8) that exceed Z-scores of 3, and 5 aCompCor [36] regressors each from eroded WM and CSF masks from (8).
10. We smoothed the fMRI data from (8) in SPM12 using an 8 mm FWHM Gaussian kernel.

### 2.5. fMRI Contrasts

Due to the constraints associated with scientific research in a very rare population, this study is significantly underpowered by modern neuroimaging standards [37,38]. With this constraint in mind, we utilized more liberal statistical thresholding to ensure we adequately describe our data. As with all studies with small samples or small populations, these results should be interpreted with caution.

Using SPM12, we analyzed participants’ BOLD activity while they performed our spatial and control tasks. In individualized first-level models, we contrasted the BOLD signal measured while participants performed the spatial task against that measured while participants performed the control task. This model included data from both preflight and postflight timepoints. We characterized each task with three regressors, one for each phase (i.e., camera moving, challenge, and post-challenge wait phases) present per trial and all phases were weighted uniformly. Therefore, each run included 6 task-based regressors and 23+ nuisance regressors from preprocessing step (9). The model included two runs per participant per timepoint. The spatial–control contrast maps were analyzed in two different second-level analyses. The first analysis was set to identify brain regions with greater BOLD activity in the spatial task than in the control task. We used a voxelwise mass univariate one-sample t-test design on each participant’s average spatial−control contrast map computed across both preflight and postflight timepoints, thresholded at a voxel height threshold of *p* < 0.0001, and a cluster extent threshold of *K_E_* > 100. The clusters identified from this analysis were used as regions of interest (ROIs) in an orthogonal analysis in which we computed the average change in spatial–control BOLD activity between preflight and postflight timepoints for each ROI using paired samples t-tests with Bonferroni correction. Additionally, we performed an exploratory whole-brain voxelwise mass univariate analysis to complement the ROI-based analysis and also to identify regions of the brain in which there was a difference in the spatial–control BOLD activity between preflight and postflight timepoints. This analysis was thresholded at a voxel height threshold of *p* < 0.0001, and a cluster extent threshold of *K_E_* > 50.

### 2.6. fMRI Correlational Analyses

We performed two correlational analyses to attempt to identify the contribution of artifactual and performance-based sources of variance. In the first analysis, we quantified the contribution of structural segmentation errors and the resultant changes in tissue concentrations at any given voxel in normalized MNI space. We previously identified that the typical change in brain position within the skull seen in astronauts between preflight and postflight time points can produce salient changes in local tissue volumes, a significant proportion of which are artifactual; these errors are produced by misclassification of different brain tissue during segmentation [39]. These tissue segmentation errors may result in certain voxels having a different concentration of grey matter between postflight and preflight and could therefore produce different BOLD responses. Although our BOLD analyses were performed on intra-run spatial–control contrasts, and theoretically self-control for timepoint-related structural changes, it is still possible that a change in the pattern of grey matter concentrations introduced some bias to our analyses investigating the change in BOLD activity between preflight and postflight time points. To investigate this possibility, we calculated the spatial correlation between voxelwise changes in BOLD activity and voxelwise changes in grey matter concentration for each individual. We also performed the ROI-based analysis of changes in BOLD activity from preflight to postflight with contrast maps residualized against grey matter concentration changes.

The second analysis was performed to identify any association between changes in behavioral performance with changes in BOLD activity between postflight and preflight timepoints. For each ROI identified in our timepoint-agnostic spatial–control analysis, we computed the Pearson correlation between participants’ change in behavioral accuracy and the change in spatial–control BOLD contrast between preflight and postflight timepoints. We again performed the ROI-based analysis of changes in BOLD activity, utilizing the grey-matter-residualized data as well as accounting for changes in behavioral performance. We evaluated the effect of spaceflight in this regression model as modeled where changes in behavioral performance would have been zero, i.e., the effects of spaceflight in this model are what would be expected with no practice effects.

## 3. Results

**Figure 2:**
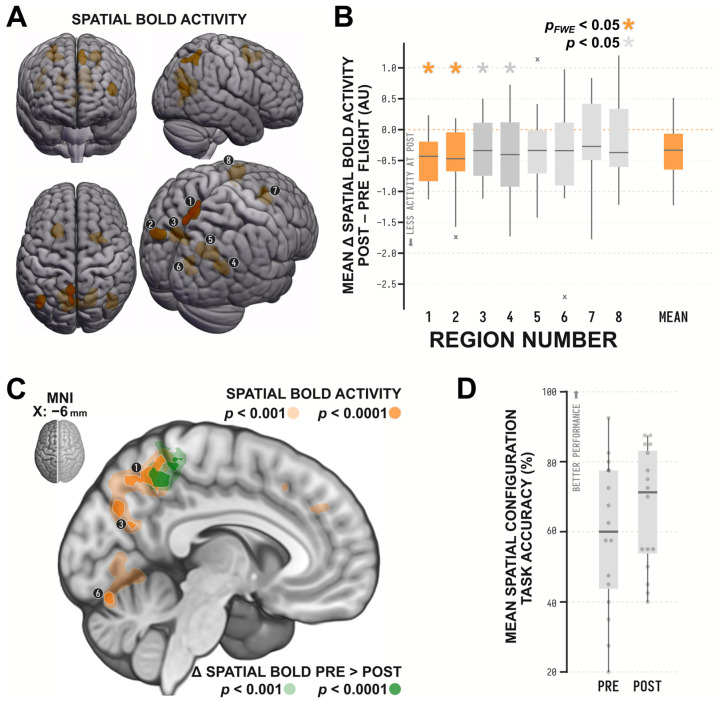
Panel (**A**) depicts the eight brain regions with increased BOLD activity in astronauts while they performed the spatial configuration task. Panel (**B**) depicts the change in this spatial BOLD activity in each of the marked ROIs after a typical mission onboard the ISS as boxplots, where x indicates outliers and asterisks flag statistically significant effects. Regions 1 and 2, localized to the precuneus and left angular gyrus, respectively, exhibited statistically significant reductions in BOLD activity during a spatial task after spaceflight. Table 1 reports region labels for the numbered ROIs as well as associated statistics for these effects. Regions are numbered based on descending statistical significance, as reported in Table 2. Panel (**C**) depicts a sagittal slice at MNI X −6 mm, with the spatial bold activity also depicted in Panel A shown in orange, and the change in spatial BOLD activity from preflight to postflight is shown in green. Panel (**D**) depicts the behavioral performance of participants on the spatial configuration task at preflight and postflight.

To explore changes in brain activity, we first identified the brain regions in astronauts that exhibited salient increases in BOLD activity in the spatial task as compared to the control task, averaged across preflight and postflight timepoints. In Table 1 and Figure 2A, we characterize the widespread network of brain regions identified in this analysis. These regions include the retrosplenial complex, located on the anterior bank of the parietooccipital fissure, and the precuneus. Both of these regions are commonly implicated in spatial orientation and navigation [23,40,41,42] and have been shown to be strongly engaged with this particular spatial task [21], along with the activity in the medial lingual gyrus also identified in our analysis. We additionally identified bilateral clusters of BOLD activity in the angular gyri, a cluster in the right frontal eye fields, and a cluster in the laterally proximal left premotor/supplementary motor region, brain regions that are also commonly implicated in spatial processing and attention [24].

With these regions of interest (ROIs) delineated, we then computed the change in spatial BOLD activity within each region between the preflight and postflight time points. This analysis, detailed in Table 2 and Figure 2B, identified significant decreases in spatial BOLD activity in the precuneus and in the left angular gyrus ROI. The decreases in the remaining ROIs did not reach statistical significance, but the mean effect across all ROIs indicated that spatial BOLD activity was generally decreased (*t*_15_ = −3.259, *p* = 0.005). A complementary exploratory whole-brain voxelwise analysis did not identify any other regions with spatial BOLD activity altered with spaceflight, only detecting a single cluster with decreased activity (*K_E_* = 67, cluster *p_FWE_* = 0.028, peak *t* = 5.19, peak *p_FWE_* = 0.067, at MNI coordinates −6, −52, 50) that overlapped heavily with the precuneus ROI (i.e., ROI #1; Figure 2C), providing some validation that this finding is not completely driven by our ROI selection procedure. We performed additional analyses (included in Table 2) to ensure that the change in spatial BOLD activity that we are attributing to spaceflight was not driven by the slight changes in behavioral performance on the spatial task—which could be attributed to practice effects, nor changes in apparent or artifactual tissue concentrations due to the changes in cerebrospinal fluid distributions resulting from spaceflight [39].

Astronauts performed well on the spatial configuration task in the MRI (Figure 2D), with a mean (SD) accuracy of 58.91 (21.37)% at preflight runs and 66.56 (17.27)% at postflight runs, representing only a marginal increase in performance (*t*_15_ = 2.051, *p* = 0.058). Astronauts’ performance at both preflight (*t*_15_ = 1.088, *p* = 0.294) and postflight (*t*_15_ = 0.427, *p* = 0.676) was similar to comparable performances we observed in healthy young adults (mean accuracy of 64.72%) in our previous research [21]. Importantly, correlations between these changes in performance and changes in BOLD activity within each ROI were weak (−0.317 < *rs* < 0.171) and not statistically significant (*ps* ≥ 0.161). Similarly, whole-brain voxelwise associations between changes in grey matter concentration and changes in BOLD activity at the subject level were weak, with *rs* ranging between −0.102 and 0.237, and as a set did not significantly differ from a null association (*M* (*SD*) = 0.001 (0.091), *t*_15_ = 0.028, *p* = 0.978). The reduction in activity we identified in the precuneus ROI remained after accounting for both of these sources of error (*t*_14_ = −3.518, *p* = 0.003), whereas the activity decrease identified in the left angular gyrus was attenuated such that it no longer survived correction for multiple comparisons (*t*_14_ = −2.371, *p* = 0.033). Overall, this indicates that practice effects and changes in grey matter distribution within the brain are not the sole mechanisms driving the changes in brain activity due to spaceflight that we described earlier.

## 4. Discussion

Teleoperation tasks, in which astronauts remotely control a machine or perform a docking maneuver, are challenging tasks that heavily rely on the operator’s spatial skills [43]. Among those skills, the ability to mentally represent the environment, and the spatial relationships between objects within it, is crucial for performing spatial tasks accurately [22]. Here, we asked astronauts to perform a task that required them to integrate multiple viewpoints into a coherent mental representation of their surroundings and use that representation to infer their location from a view within the environment [21]. Brain activity recorded while astronauts performed this task before and after spaceflight revealed two important findings. First, we identified a general reduction in the average spatial BOLD activity at the postflight timepoint across the network of spatial processing regions within the brain. Second, we identified that this effect was most salient in the precuneus and left angular gyrus; importantly, the reduction in spatial BOLD activity in the precuneus remained statistically significant after accounting for changes in behavioral performance and changes in grey matter concentration from preflight to postflight.

The precuneus and the neighboring posterior medial parietal cortex are characterized as the structural core of the cerebral cortex due to their dense interconnectedness and topographic centrality [44,45]. Cognitively, the precuneus has been associated with visuospatial imagery, episodic memory retrieval, and self-referential processing [40]. Numerous subregions within the precuneus show structural and functional connections with the angular gyrus and the nearby retrosplenial complex and posterior cingulate cortex [46]. Of these networked regions, the angular gyrus is known to be engaged in left–right discrimination as well as directing spatial attention, particularly motion-driven and bottom-up attention allocation [47,48]. On the other hand, the retrosplenial complex has been implicated in spatial layout encoding, integration, and retrieval [23,42,49].

Previous research on the effects of spaceflight on cognition and brain function has often attributed the observed difficulties in spatial processing to spaceflight-related vestibular adaptation or dysfunction [14,50]. In the non-astronaut population, vestibular dysfunction is known to produce a surprisingly broad array of cognitive and behavioral changes [51,52], and Reschke and Clément [14] made a compelling case that changes in vestibular processing may be the ultimate cause of the spatial processing difficulties reported in astronauts. Considering the general observation that many forms of spatial processing rely on multimodal sensory information that includes vestibular processing [16,29,53], together with the reduction in vestibular weight in visual-vestibular sensory weighting due to spaceflight [12,14], the alterations in vestibular processing are a very likely and reasonable cause of many observed changes in spatial cognition in astronauts.

However, with respect to the present results, vestibular processes are not routinely associated with precuneus structure and function; most existing evidence supports indirect precuneal involvement in some clinical conditions, e.g., epilepsy and vestibular migraine [54,55,56]. Characterizations of the extended vestibular processing network in the brain typically do not include the precuneus [57,58], nor does it appear to be affected by the vestibular reweighting seen in astronauts after spaceflight [13]. In our experimental timeline, in which astronauts were tested approximately 12 days after landing, we expected that vestibular readaptation to the terrestrial environment should have occurred [12,59,60]. Furthermore, the involvement of vestibular processes, particularly motion-related processes, in fMRI experiments in general is thought to be relatively small [61]. In our experiment, and typically in fMRI studies of cognition, participants lay in the MRI supine, with their head surrounded by padding to prevent head movement, and are instructed to lie as still as possible. (Even small head movements can produce a significant amount of noise and spurious signal in fMRI data [62,63]). This renders the typical vestibular contribution in fMRI studies of spatial cognition to be far lesser than in ambulatory conditions [29]. These factors lead us to believe that the reductions in BOLD activity in our task paradigm are more indicative of changes in the manner in which spatially relevant information is being mentally manipulated by astronauts after their spaceflight experience and are not directly driven by changes in vestibular processing.

This paradigm is also consistent with astronauts’ reports of changes in the way they navigate and mentally represent their surroundings for the purposes of navigation. Many astronauts have reported to us that their sense of direction is unreliable when working in atypical orientations or transiting between ISS modules with inconsistent internal orientations. As reported previously in the literature [64], astronauts mitigate these issues by adopting new reorientation and wayfinding strategies. These strategies typically involve visuospatial landmark-based reorientation and/or behavioral patterns, e.g., “when exiting the cupola, I put my back to the hatch and Node 1 will be on the left”, or “always pitch belly-down when entering Nakua”. Although both of these examples make use of spatial information, they do not require a coherent spatial representation of the environment to be successfully utilized, nor do they indicate an intuitive sense of direction. We expect that the more that astronauts make use of such strategies, the less they would recruit processes and brain regions that are specialized in processing spatial information (as experienced on Earth), particularly those with an involvement in generating and using a mental representation of the spatial environment. Our findings support this conclusion, as we showed a general reduction in the activity of the brain regions involved in spatial processing in astronauts at postflight. This suggests that after their spaceflight, astronauts might have performed our spatial task in a manner more similar to a generic working memory task. Of these affected brain regions, the engagement of the precuneus in our spatial task was most strongly impacted by spaceflight. Like many brain regions, the precuneus has been implicated in a handful of behavioral and cognitive functions, but it is reliably associated with spatially guided behavior, integrating egocentric and allocentric spatial information, and imagining movement [40,65,66,67]. The lowered levels of activity seen from the precuneus after spaceflight would indicate that astronauts were relying less on these uniquely spatial processes to solve spatial problems.

Neuroimaging studies in astronauts have a handful of known limitations, of which the present study is not immune. The small size and restricted access to and availability of this population left the present study underpowered by current neuroimaging standards [37,38]. Furthermore, spaceflight is a unique and complex experience that renders the mechanistic inferences of the cognitive effects of spaceflight as speculative. We have attempted to minimize the influence of one of the known threats to interpretive validity in neuroimaging studies in astronauts: the change in segmentation error bias driven by the redistribution of cerebrospinal fluid in the skull that we have described previously [39]. However, there are other potential threats to interpreting BOLD signals in astronauts after spaceflight, such as the changes in brain perfusion observed after a head-down bed rest study [68], a paradigm commonly employed as a spaceflight analog. Due to both known and unknown threats to the validity of our experiment, we recommend that these findings be treated as preliminary and interpreted with caution.

In conclusion, our findings provide an initial characterization of the neural mechanisms of a cognitive adaptation to spaceflight, suggesting astronauts rely less on the cognitive strategies and neural resources they used at preflight time points, resources that are presumably specialized for terrestrial spatial processing. These preliminary findings are particularly interesting because they extend beyond the known sensorimotor changes associated with spaceflight [14,50,69,70], which include postural instability, motor impairments, and visual illusions, and instead, implicate an alteration of the cognitive processes involved in computing and integrating spatial information from multiple viewpoints. We drew this conclusion partially from phenomenological reports of astronaut’s experiences in microgravity, but it remains to be determined how those changes take place in space during the course of their mission. Furthermore, the timeline and permanence of this effect after astronauts return to Earth, and its relevance for daily tasks in astronauts’ personal or professional lives, remain to be established. Future missions to Mars or the Moon with spatial tasks (e.g., teleoperative tasks, or driving a rover) that may need to be performed shortly after transitions between gravity levels, where spatial processing will be particularly impaired, may be vulnerable to some of the changes in the neural mechanisms we identified here. Such tasks may be more reliably performed by astronauts if the procedures and training provided minimize reliance on explicitly spatial language and processes and lean more heavily into behavior-oriented and semantic task paradigms. Alternatively, automated or software-assisted countermeasures may best focus on offloading demands for astronauts to wayfind, track non-visible landmarks, and judge angles and distances, as these spatial processes are likely affected by spaceflight.

## Figures and Tables

**Table 1:** Brain regions with increases in BOLD activity during a spatial task, above that seen in the control task. ROI #s provided for cross-reference with Table 2 and Figure 2, order determined by descending statistical significance in Table 2. Cluster extents (*K_E_*) are reported in 2 mm isotropic voxel counts, coordinates reported in MNI space.

	Cluster			Peak				
ROI #	Label	*K_E_*	*p_FWE_*	*t*_15_	*p_FWE_*	x	y	z
1	Precuneus	134	0.001	5.83	0.448	−8	−56	54
				5.61	0.553	−6	−66	50
				5.37	0.673	−8	−52	44
2	Left Angular Gyrus	128	0.001	6.45	0.229	−42	−64	12
				5.69	0.515	−44	−74	18
3	Left Dorsal Retrosplenial Complex or Posterior Cingulate Cortex	376	<0.001	8.01	0.036	−16	−58	20
				7.26	0.087	−10	−74	34
				5.80	0.462	−4	−70	26
4	Right Angular Gyrus	198	<0.001	7.49	0.066	42	−64	20
				5.86	0.435	40	−72	36
				5.78	0.451	44	−76	26
5	Right Dorsal Retrosplenial Complex or Posterior Cingulate Cortex	287	<0.001	9.10	0.010	18	−66	26
				5.90	0.416	20	−56	14
6	Lingual Gyrus	117	0.001	5.86	0.433	0	−70	6
				5.66	0.526	12	−72	2
7	Right Frontal Eye Fields	188	<0.001	6.21	0.301	26	10	50
				6.08	0.347	36	4	58
				5.78	0.469	16	12	54
8	Left Premotor or Supplementary Motor Cortex, BA6	217	<0.001	7.13	0.103	−20	22	56
				6.75	0.160	−18	12	54
				5.70	0.511	−20	22	38

**Table 2:** The effects of spaceflight (postflight–preflight) on spatial BOLD activity. ROI # provided for cross-reference with Figure 2 and Table 1. We accounted for changes in grey matter (GM) concentration by residualizing each subject’s voxelwise contrast map against their voxelwise GM concentration change map. * indicates an effect that is significant at *α* < 0.05, *** indicates an effect that is significant at a Bonferroni-corrected (for 8 comparisons) FWE threshold of *α* < 0.006.

	Effect of Spaceflight on Spatial BOLD			Correlation with Change in Spatial Performance			Effect Accounting for Changes in GM Concentration			Effect Accounting for Changes in GM Concentration and Performance		
ROI #	*t*_15_	*p*	*sig*	*r*_14_	*p*	*sig*	*t*_15_	*p*	*sig*	*t*_14_	*p*	*sig*
1	−4.959	<0.001	***	−0.317	0.232		−4.542	<0.001	***	−3.518	0.003	***
2	−3.427	0.004	***	−0.307	0.248		−3.291	0.005	***	−2.371	0.033	*
3	−2.366	0.032	*	−0.079	0.770		−2.145	0.049	*	−1.698	0.112	
4	−2.243	0.040	*	−0.368	0.161		−2.164	0.047	*	−1.296	0.216	
5	−2.020	0.062		−0.151	0.576		−1.760	0.099		−1.256	0.230	
6	−1.974	0.067		−0.118	0.662		−1.935	0.072		−1.466	0.165	
7	−1.081	0.297		0.129	0.634		−0.809	0.431		−1.004	0.332	
8	−1.045	0.313		0.171	0.527		−0.690	0.501		−0.983	0.342	

## Data Availability

To protect participant privacy, the data that support these findings are not openly available.
